# Pneumococcal Detection and Bacterial Co-Detection in Children After COVID-19: A Two-Year Multiplex PCR Study

**DOI:** 10.3390/biomedicines14061381

**Published:** 2026-06-18

**Authors:** Loredana Stavăr-Matei, Lavinia Țocu, Aurel Nechita, Luiza Camelia Nechita, Oana Mariana Mihailov, Florentin Dimofte, George Țocu

**Affiliations:** 1 Faculty of Medicine and Pharmacy, Research Center in the Medical-Pharmaceutical Field, “Dunărea de Jos” University, 800008 Galati, Romania; mateiloredana23@yahoo.com (L.S.-M.); lavinia.tocu@ugal.ro (L.Ț.);; 2 “Sf. Ioan” Children’s Emergency Hospital, 800487 Galati, Romania; 3 “Sf. Apostol Andrei” County Emergency Clinical Hospital, 800578 Galati, Romania; 4 “Sfântul Spiridon” Clinical Hospital of Pneumophthisiology, 800552 Galati, Romania

**Keywords:** Streptococcus pneumoniae, Haemophilus influenzae, multiplex PCR, bacterial co-detection, post-COVID-19, immunity debt, community-acquired pneumonia, children

## Abstract

**Background**: Non-pharmaceutical interventions during the COVID-19 pandemic altered respiratory pathogen circulation, and a bacterial rebound followed once restrictions were lifted. We describe pediatric pneumococcal respiratory infections and their bacterial co-detections in the immediate post-pandemic period. **Methods**: We retrospectively analyzed respiratory specimens from children aged 0–18 years tested with a multiplex real-time PCR panel (Allplex Respiratory Panel, Seegene, Seoul, South Korea; seven bacterial pathogens) restricted to this predefined bacterial spectrum at a tertiary pediatric hospital in Galați, Romania, during 2022 and 2023. A total of 2546 panels were performed in 2022 and 3250 in 2023, allowing pneumococcal positivity rates to be calculated. Proportions are reported with Wilson 95% confidence intervals; associations were tested with Pearson chi-square and Fisher exact tests in SPSS v.23. **Results**: Children with detected Streptococcus pneumoniae rose from 100 to 415, corresponding to a rise in pneumococcal positivity from 3.9% (100/2546) to 12.8% (415/3250). Among the positive children, pneumococcus–Haemophilus influenzae co-detection increased from 33.0% to 45.1% (odds ratio 1.63, 95% CI 1.02–2.61; *p* = 0.029), while pneumococcus alone fell from 60.0% to 50.1%. Boys, urban residence, and early childhood predominated, and community-acquired pneumonia diagnoses rose from 61 to 214. No profile–demographic association reached significance (panel–residence 2023, *p* = 0.063). **Conclusions**: A post-pandemic rise in pediatric pneumococcal detections and increasing H. influenzae co-detection were observed, supporting syndromic multiplex PCR in rapid pediatric diagnostics and antimicrobial stewardship.

## 1. Introduction

Acute respiratory tract infections remain a leading cause of morbidity and healthcare use in children, and bacterial pathogens contribute to their most severe presentations [1,2]. Streptococcus pneumoniae is the foremost bacterial cause of community-acquired pneumonia, otitis media, sepsis, and meningitis in children, with the greatest burden below five years of age [3,4,5]. The pneumococcus colonizes the nasopharynx of many healthy children, and this carriage is both the reservoir for transmission and the first step toward mucosal and invasive disease [6,7,8]. Demographic and environmental determinants, including age, sex, and living conditions, modulate the risk of pediatric respiratory infection [9].

The COVID-19 pandemic and the non-pharmaceutical interventions used to contain it—masking, distancing, school closures, and reduced mixing—markedly disrupted respiratory pathogen circulation. Early in the pandemic, a steep decline in invasive disease due to *S. pneumoniae*, Haemophilus influenzae, and Neisseria meningitidis was documented across many countries [10,11]. This reduced exposure is thought to have generated an “immunity debt”, leaving a larger susceptible population once interventions were relaxed [12,13]. The lifting of restrictions was followed by an abrupt resurgence of respiratory infections, frequently exceeding the pre-pandemic levels [14,15,16]. A comparable post-pandemic effect was reported for pneumococcal acute otitis media, antibiotic resistance, and vaccination uptake in children [17]. Similar pandemic-related shifts in the burden and antimicrobial susceptibility of pediatric bacterial infections have also been described for other pathogens in the same region of southeastern Romania, underscoring the value of monitoring local resistance patterns across the pandemic transition [18].

This shifting epidemiology has been particularly evident in children, with overlapping outbreaks and a rise in bacterial co-detections [14,15]. The interaction between *S. pneumoniae* and *H. influenzae* is of special interest: the two organisms share the nasopharyngeal niche, and their relationship spans synergy and competition, with consequences for colonization density and disease expression [19,20,21,22,23,24,25]. Co-colonization has been associated with enhanced pneumococcal-specific antibody responses [26], while in other settings, H. influenzae competes with and even predominates over the pneumococcus [27,28]. A positive association between H. influenzae and S. pneumoniae nasopharyngeal carriage has been reported in children irrespective of HIV status [29]. Understanding the relative frequency of pneumococcus alone versus combined detections therefore has clinical and ecological relevance, especially in the conjugate-vaccine era [21,30,31,32,33,34].

Conventional culture-based diagnosis is limited by slow turnaround, reduced sensitivity after antibiotic exposure, and difficulty distinguishing colonization from infection [28]. Syndromic multiplex real-time PCR panels detect multiple pathogens directly from respiratory specimens, supporting earlier targeted therapy, shorter hospitalization, and improved antimicrobial stewardship [35,36,37,38,39,40]. Rapid molecular results can reduce unnecessary antibiotic prescriptions and improve treatment appropriateness in children with respiratory infection [41,42,43]. A rapid PCR-based approach has proven useful specifically for diagnosing pneumococcal infection in the context of bacterial co-infection in children [44].

In Romania, pneumococcal conjugate vaccination entered the national program in 2017, yet a substantial residual burden of pneumococcal disease persists, and modeling continues to evaluate higher-valent vaccines [45,46]. The disease also carries psycho-emotional consequences for children and families [47]. Against this background, the present study describes pediatric pneumococcal respiratory infections and their bacterial co-detections during the two years immediately following the relaxation of pandemic restrictions (2022–2023), using a multiplex respiratory PCR panel at a tertiary pediatric center, and explores associations between the bacterial profile and key demographic and clinical variables. By design, the study was limited to the seven bacterial pathogens included in the multiplex panel; viral agents, fungi, and opportunistic or conditionally pathogenic organisms that may have emerged after the pandemic were outside its predefined scope and were not investigated.

## 2. Materials and Methods

### 2.1. Study Design and Population

We conducted a retrospective observational study at the “Sf. Ioan” Clinical Emergency Hospital for Children in Galați, Romania, including pediatric patients aged 0–18 years investigated for suspected respiratory infection during the post-pandemic period of 2022 and 2023. Patients were eligible if a respiratory specimen had been tested with the multiplex PCR respiratory panel and S. pneumoniae was detected, alone or combined with other panel bacteria. The total number of multiplex respiratory PCR panels performed during the study period was retrieved from the laboratory information system: 2546 panels in 2022 and 3250 in 2023. These denominators allowed pneumococcal positivity rates to be calculated for each year. Data were retrieved from the hospital electronic archive and processed with Microsoft Office 365 and IBM SPSS Statistics v.23. A positive PCR result was interpreted in conjunction with the clinical presentation recorded by the attending physician rather than in isolation. Because PCR detects nucleic acid and cannot by itself separate true infection from colonization or specimen contamination, cases were considered clinically relevant when the molecular detection was accompanied by a compatible respiratory syndrome; supporting parameters such as inflammatory markers and chest imaging were used by the treating clinicians when available, although these were not systematically recorded in the retrospective archive and could not be analyzed uniformly.

### 2.2. Specimen Collection and Multiplex PCR Testing

Respiratory specimens consisted of nasopharyngeal/pharyngeal swabs and sputum, processed on the Allplex platform (Allplex Respiratory Panel, Seegene, Seoul, Republic of Korea) using the respiratory bacterial panel, a multiplex real-time PCR assay that detects and differentiates seven bacterial pathogens responsible for the most frequent respiratory tract infections: Bordetella parapertussis (BPP), Bordetella pertussis (BP), Chlamydophila pneumoniae (CP), Haemophilus influenzae (HI), Legionella pneumophila (LP), Mycoplasma pneumoniae (MP), and Streptococcus pneumoniae (SP). The assay identifies pathogen DNA directly from the specimen, providing a rapid molecular result that can guide etiology-directed therapy [35,37,44]. The panel was deliberately restricted to this predefined set of seven bacterial species; viruses, fungi, and opportunistic or conditionally pathogenic organisms were not part of the assay and were therefore not assessed. The great majority of specimens were nasopharyngeal/pharyngeal swabs, with only a small minority of sputum samples; both were analyzed with the same molecular assay and were pooled because the retrospective archive did not allow for a sufficiently large, separately powered analysis of the sputum subgroup. All children in the study cohort had received pneumococcal and Haemophilus influenzae type b vaccination in accordance with the Romanian national immunization schedule.

### 2.3. Variables

Variables included sex, area of residence (urban/rural), age group (early childhood, preschool, school age, adolescence), specimen type (nasopharyngeal/pharyngeal swab vs. sputum), clinical diagnosis at presentation, and the respiratory panel profile (pneumococcus alone vs. pneumococcus combined with one or more additional panel bacteria, predominantly H. influenzae).

### 2.4. Statistical Analysis

Categorical variables were summarized as counts and as proportions of the pneumococcus-positive population for the corresponding year. Because the total number of respiratory PCR panels performed each year was available (2546 in 2022 and 3250 in 2023), the pneumococcal positivity rates were also computed as the proportion of all tested children with a positive S. pneumoniae result. Wilson score 95% confidence intervals (CIs) were computed for proportions. Associations between categorical variables were examined with the Pearson chi-square test; when more than 20% of cells had an expected count below five, the Fisher exact test was applied. To quantify the between-year change in H. influenzae co-detection, an odds ratio (OR) with a 95% CI was calculated from the 2 × 2 table of co-detection versus pneumococcus alone; because only two years were compared, this represents a difference between two groups rather than a trend across multiple ordered time points, and the associated *p*-value was derived from the corresponding chi-square test. A two-sided *p* < 0.05 was considered significant.

## 3. Results

### 3.1. Overall Frequency and Bacterial Co-Detection

**Figure 1:**
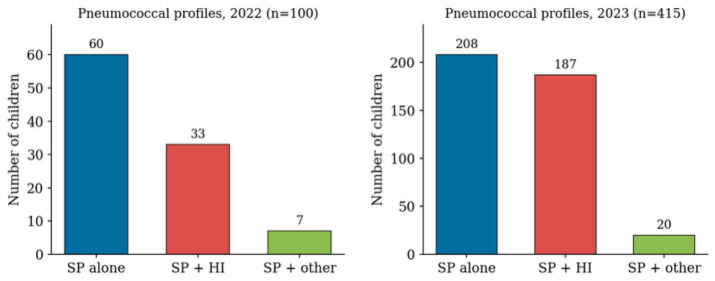
Number of children with Streptococcus pneumoniae detected by the multiplex respiratory PCR panel, by bacterial profile, in 2022 (*n* = 100, (**left**)) and 2023 (*n* = 415, (**right**)). SP, S. pneumoniae; HI, H. influenzae.

Across the two study years, the number of pediatric pneumococcal detections increased markedly. In 2022, S. pneumoniae was identified in 100 children: alone in 60 (60.0%; 95% CI 50.2–69.1), with H. influenzae in 33 (33.0%; 95% CI 24.6–42.7), and with other bacteria in 7 (7.0%), the latter including triple combinations also involving B. parapertussis. In 2023, the total rose to 415 children: pneumococcus alone in 208 (50.1%; 95% CI 45.3–54.9), pneumococcus–H. influenzae in 187 (45.1%; 95% CI 40.3–49.9), and pneumococcus with other bacteria in 20 (4.8%) (Table 1, Figure 1). When referred to the total number of panels performed (2546 in 2022 and 3250 in 2023), the pneumococcal positivity rate rose from 3.9% (100/2546; 95% CI 3.2–4.7) in 2022 to 12.8% (415/3250; 95% CI 11.7–14.0) in 2023. This increase therefore persisted after accounting for testing volume and did not simply reflect a larger number of panels ordered. Among the positive children, the relative weight of H. influenzae co-detection rose from about one third to nearly one half of cases. This between-year difference in the co-detection profile was statistically significant (OR 1.63; 95% CI 1.02–2.61; chi-square *p* = 0.029).

### 3.2. Distribution by Sex

**Figure 2:**
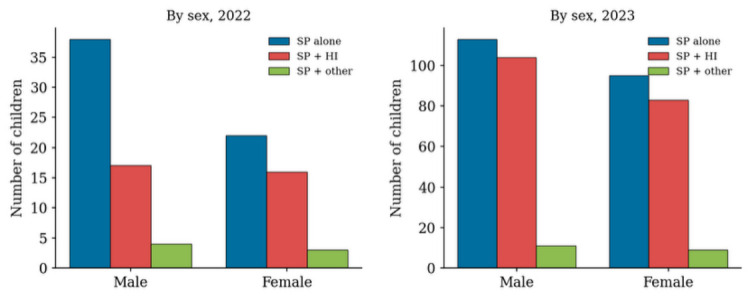
Number of children with S. pneumoniae detected alone, by sex, in 2022 (**left**) and 2023 (**right**). Only measured counts for the SP-alone profile are shown; sex-disaggregated counts for the co-detection profiles were not separately recorded and are not displayed. SP, S. pneumoniae.

In both years, boys were more frequently affected than girls. In 2022, pneumococcus alone was detected in 38 boys versus 22 girls. In 2023, the male predominance persisted, with 113 boys versus 95 girls among children with pneumococcus alone. Sex-disaggregated counts for the co-detection profiles were not separately recorded and are therefore not reported (Figure 2). A male predominance in pediatric respiratory pathogen detection is consistent with the broader literature on sex differences in childhood respiratory infection [9].

### 3.3. Distribution by Area of Residence

In both 2022 and 2023, more cases originated from urban areas, both for pneumococcus alone and for pneumococcus combined with H. influenzae, consistent with observations from earlier periods at the same center. In 2023, 249 of 415 children (60.0%; 95% CI 55.2–64.6) were from urban areas and 166 (40.0%) from rural areas (Table 2).

### 3.4. Distribution by Age Group

**Figure 3:**
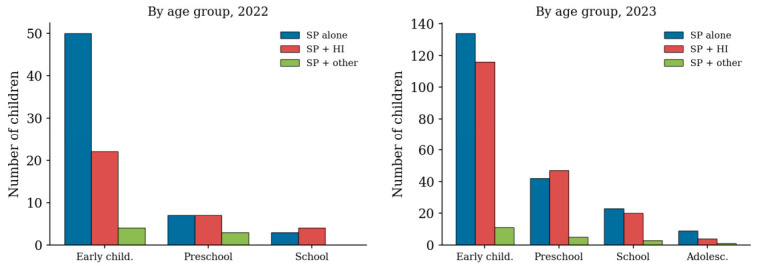
Pneumococcal detections and bacterial co-detection by age group, 2022 (**left**) and 2023 (**right**). In 2022, three age strata were recorded; in 2023, four strata including adolescence.

Early childhood was the most affected age group, both for pneumococcus alone and for bacterial co-detection. In 2023, of the 415 children, 261 (62.9%; 95% CI 58.1–67.4) were in early childhood, 94 (22.7%) preschool, 46 (11.1%) school age, and 14 (3.4%) adolescents (Table 3, Figure 3). This distribution agrees with the well-described peak of pneumococcal and Haemophilus carriage and disease in the first years of life [6,20,21].

### 3.5. Specimen Type and Pneumonia Diagnoses

Pneumococcus, alone and in co-detection, was identified in over 90% of cases from nasopharyngeal/pharyngeal specimens, with the remainder from sputum. Because nasopharyngeal/pharyngeal swabs accounted for the great majority of specimens, the detection profile essentially reflects this single specimen type, the sputum samples being too few to support a separate comparison. The detections also showed a clear seasonal pattern: most specimens were collected during the cold season, in the autumn–winter months of October, November, and December, consistent with the expected winter peak of pediatric respiratory infections. The number of children with community-acquired pneumonia rose from 61 in 2022 to 214 in 2023. For this study, community-acquired pneumonia comprised both clinically suspected cases (compatible clinical presentation recorded by the attending physician) and radiologically confirmed cases (consolidation on chest radiography); the two categories were grouped together because the retrospective archive did not consistently distinguish them. This increase paralleled the overall rise in pneumococcal detections [15,30,48,49].

### 3.6. Statistical Associations

In 2022, contingency analyses between the respiratory panel profile and age group, specimen type, and area of residence, and between clinical diagnosis and age group, all violated the expected-count assumptions; the Fisher exact test was therefore used. None reached significance: diagnosis versus age group (Fisher exact = 18.186, *p* = 0.528); panel versus age group (Fisher exact = 7.917, *p* = 0.180); panel versus specimen (Fisher exact = 0.746, *p* = 0.840); panel versus residence (Fisher exact = 2.791, *p* = 0.449) (Table 4).

In 2023, the larger sample permitted more robust testing. Panel versus age group was non-significant (Fisher exact = 4.057, *p* = 0.644), as was panel versus specimen type (Fisher exact = 5.196, *p* = 0.223). For panel versus area of residence, the chi-square assumptions were met, and the Pearson chi-square was 5.530 (df = 2, *p* = 0.063); because this value did not cross the predefined 0.05 threshold, the association was non-significant (Table 4).

In addition, the between-year rise in H. influenzae co-detection among positive cases was significant: the odds of SP–HI co-detection (versus pneumococcus alone) were higher in 2023 than in 2022 (OR 1.63; 95% CI 1.02–2.61); the corresponding chi-square test confirmed that this difference between the two years was statistically significant (*p* = 0.029). Because only two years were compared, this is a between-year difference rather than a temporal trend.

## 4. Discussion

In this two-year, single-center study, pediatric pneumococcal respiratory detections increased from 2022 to 2023, with the pneumococcal positivity rate rising from 3.9% to 12.8% after accounting for the number of panels performed each year, accompanied by a significant rise in H. influenzae co-detection (OR 1.63; 95% CI 1.02–2.61; *p* = 0.029); this association should nonetheless be interpreted with caution, as the lower bound of the confidence interval lies only marginally above 1, indicating a modest effect size that warrants confirmation in larger studies. The timing aligns with the widely reported post-pandemic resurgence of respiratory pathogens attributed to “immunity debt”: the accumulation of immunologically naïve children during periods of low circulation, followed by a surge once non-pharmaceutical interventions were relaxed [12,13,14]. Large surveillance datasets have documented analogous 2023 rebounds—for respiratory syncytial virus and Mycoplasma pneumoniae in particular—often exceeding the pre-pandemic levels [15,16]. Our pneumococcal findings extend this pattern to a bacterial otopathogen in a Romanian pediatric population and are consistent with our earlier observation of post-pandemic shifts in pneumococcal acute otitis media and antibiotic resistance [17].

The increasing share of pneumococcus–H. influenzae co-detection is biologically plausible. The two species share the nasopharyngeal niche and exhibit complex synergistic and competitive interactions that influence colonization density, persistence, and disease expression [19,20,21,22]. A positive co-colonization association has been reported in children irrespective of HIV status [29], and co-colonization can enhance pneumococcal-specific antibody responses [26]; conversely, in acute otitis media, H. influenzae may predominate over most pneumococcal strains [27,28]. Within this framework, the rising co-detection we observed may reflect intensified transmission and denser polymicrobial carriage as social mixing resumed, rather than a change in intrinsic species interaction. Rapid molecular detection of such co-infections is clinically valuable, as we have shown for pneumococcal infection identified in the setting of bacterial co-infection [44]. An additional consideration is vaccination status. All children in the present cohort had received pneumococcal conjugate and Haemophilus influenzae type b vaccination according to the national schedule, so the detections reported here occurred in a uniformly vaccinated population. This is relevant because conjugate vaccination reshapes the pneumococcal ecology—through serotype replacement and altered competitive balance in the nasopharynx—and can thereby influence pneumococcal–Haemophilus interactions and co-colonization [21,30]. The homogeneous vaccination background of our cohort means that the observed rise in detections and co-detections cannot be attributed to differences in vaccine exposure between the two years; it does, however, limit our ability to assess how vaccination itself shapes these patterns, which would require serotype-level data that were not available here.

The demographic pattern—male predominance, urban origin, and a peak in early childhood—is consistent with the known epidemiology of pneumococcal and Haemophilus carriage and disease in the first years of life [6,9,20], and with risk-factor analyses in pediatric respiratory infection [9]. The parallel rise in community-acquired pneumonia diagnoses (from 61 to 214) underscores the growing clinical burden and the relevance of these detections beyond simple carriage [30]. Notably, no association between the bacterial profile and age group, specimen type, or residence reached statistical significance in either year. The panel–residence comparison in 2023 was non-significant (*p* = 0.063); any possible association would need to be examined in a larger, adequately powered study.

These findings carry practical implications that, although not directly tested in the present study, are supported by external evidence. In other studies, syndromic multiplex PCR panels that identify multiple pathogens directly from respiratory specimens have been associated with reduced unnecessary antibiotic use, more appropriate therapy, and shorter hospitalization in children [35,36,38,41,42,43]; our study did not assess antibiotic prescribing or clinical outcomes and so cannot itself demonstrate a stewardship benefit. Reports of a persistent pneumococcal burden despite conjugate vaccination and the ongoing evaluation of higher-valent vaccines in Romania [45,46] likewise argue, in the wider literature, for continued molecular surveillance; serotype and susceptibility data were not collected here, so these points are presented as context rather than as findings of the present work. The wider experience of the COVID-19 pandemic in Romania, including the burden of healthcare-associated infections in hospitalized patients and the regional variability in infection prevention and control, further supports the need for strengthened, standardized surveillance systems aligned with international standards [50,51]. The psycho-emotional impact of invasive pneumococcal disease on children and parents, described elsewhere, adds a dimension that purely microbiological metrics do not capture [47].

### Limitations

Several limitations should be acknowledged. First, although the total number of panels performed each year was available and allowed positivity rates to be computed, the indications for testing may have shifted between 2022 and 2023, so changes in testing practice cannot be entirely excluded as a partial contributor to the observed increase. Second, this is a single-center retrospective study, limiting generalizability. Third, PCR detects nucleic acid and cannot by itself distinguish colonization from true infection. Fourth, serotype and antimicrobial-susceptibility data were not available, and the panel targets a fixed set of seven bacteria. Finally, sex-disaggregated counts for the co-detection profiles were not separately recorded; only measured counts are reported, and no estimated values are presented. Beyond these points, several further constraints follow from the retrospective design and the predefined assay. The multiplex panel targeted only seven bacterial species and did not include viruses, fungi, or opportunistic and conditionally pathogenic organisms, some of which became more prominent after the pandemic; the findings should therefore be read strictly within this bacterial spectrum. Specimens were predominantly nasopharyngeal/pharyngeal swabs with a small minority of sputum samples, and the two specimen types were pooled; because microbial composition can differ between sample types, a separate, adequately powered single-specimen analysis was not feasible, and the results essentially reflect upper-respiratory swabs. Supporting clinical and laboratory criteria for confirming true infection—such as C-reactive protein, chest imaging, and other inflammatory markers—were used by treating clinicians but were not recorded uniformly in the archive and could not be analyzed systematically, so the distinction between infection, carriage, and contamination relied on clinical correlation rather than a standardized case definition. Information on prior or concurrent antimicrobial therapy and on whether testing was performed for screening or for symptomatic disease was likewise incomplete. Finally, comparable panel data from the pre-pandemic or intra-pandemic periods were not available at our center, so the post-pandemic pathogen profile could not be directly compared with an earlier baseline; such a comparison would strengthen the interpretation and is a priority for future work.

## 5. Conclusions

In this Romanian pediatric cohort, pneumococcal respiratory detections increased between 2022 and 2023, with the pneumococcal positivity rate rising from 3.9% to 12.8% and a significant change in the H. influenzae co-detection profile among positive children, alongside a parallel increase in community-acquired pneumonia diagnoses. The demographic profile—male, urban, early childhood—matched established pneumococcal epidemiology, and no profile–demographic association reached significance. The most robust finding, independent of testing volume, was the 2023 shift in the co-detection mix among pneumococcus-positive children. These observations are compatible with a post-pandemic increase in pneumococcal circulation; the potential role of syndromic multiplex PCR in rapid pediatric diagnostics and antimicrobial stewardship is supported by other studies rather than demonstrated here. Prospective, multicenter studies incorporating serotyping, susceptibility, and clinical-outcome data are warranted.

## Figures and Tables

**Table 1:** Distribution of respiratory panel profiles among children with detected Streptococcus pneumoniae, 2022 vs. 2023.

Respiratory Panel Profile	2022, *n* (%)	2023, *n* (%)
S. pneumoniae alone	60 (60.0)	208 (50.1)
S. pneumoniae + H. influenzae	33 (33.0)	187 (45.1)
S. pneumoniae + other bacteria	7 (7.0)	20 (4.8)
Total with S. pneumoniae	100 (100)	415 (100)

**Table 2:** Respiratory panel profile by area of residence, 2023 (R = rural, U = urban).

Area	SP Alone	SP + HI	SP + Other	Total
Rural	87	76	3	166
Urban	121	111	17	249
Total	208	187	20	415

**Table 3:** Respiratory panel profile by age group, 2023.

Age Group	SP Alone	SP + HI	SP + Other	Total
Early childhood	134	116	11	261
Preschool	42	47	5	94
School age	23	20	3	46
Adolescence	9	4	1	14
Total	208	187	20	415

**Table 4:** Summary of statistical tests for associations between the respiratory panel profile (and diagnosis) and demographic/clinical variables for 2022 and 2023.

Year/Comparison	Test	Statistic	*p*-Value
2022: diagnosis × age group	Fisher exact	18.186	0.528
2022: panel × age group	Fisher exact	7.917	0.180
2022: panel × specimen	Fisher exact	0.746	0.840
2022: panel × residence	Fisher exact	2.791	0.449
2023: panel × age group	Fisher exact	4.057	0.644
2023: panel × specimen	Fisher exact	5.196	0.223
2023: panel × residence	Pearson χ^2^	5.530	0.063

## Data Availability

The data presented in this study are available on reasonable request from the corresponding author. The data are not publicly available due to privacy restrictions.
